# Building bridges: Connecting sport marketing and critical social science research

**DOI:** 10.3389/fspor.2022.970445

**Published:** 2022-09-16

**Authors:** Zachary Charles Taylor Evans, Sarah Gee, Terry Eddy

**Affiliations:** Department of Kinesiology, Faculty of Human Kinetics, University of Windsor, Windsor, ON, Canada

**Keywords:** consumer research, commercialization, circuit of culture, consumer culture theory, corporate partnerships

## Abstract

Recently, sport management scholars have called for researchers to critically evaluate the ways in which research questions and resulting contributions truly disrupt what is known, how it is known, why it is important to know, and for whom. Historically, sport marketing research has adapted traditional research approaches from the parent marketing discipline to sport. Yet, sport is a constantly evolving social and cultural phenomenon and a reliance on conventional theories, concepts, and methods can serve to crystalize the discourse in sport marketing in ways that may limit knowledge production. Responding to this call, we believe that sport marketing research has much to gain from engaging with critical social science assumptions, worldviews, and perspectives to examine complex issues in sport. We position this paper as a starting point for advancing the field of sport marketing in meaningful and impactful ways by offering two research propositions, each accompanied by four actional recommendations. We employ a particular focus on the marketing campaigns that activate and promote corporate partnerships in sport to frame our two propositions, which discuss (1) consumer culture theory and (2) the circuit of culture as two important frameworks that begin to build bridges between sport marketing and critical social science.

## Introduction

Researchers of sport and sport-related groups are being nudged by institutions, funding bodies, and publication “gatekeepers” (e.g., editors and reviewers) to justify the value of their work in ways that move beyond addressing a gap in the literature. While “gap-spotting” is a common way to formulate research questions from existing literature, Sandberg and Alvesson (1) claim that “it does not actively challenge the assumptions underlying existing theory” (p. 33). Alternatively, they suggest that problematization leads to more innovative and novel research questions that “disrupt the reproduction and continuation of an institutionalized line of reasoning” [(1), p. 32]. Recently, Stenling and Fahlén (2) call for sport management researchers (broadly interpreted) to consider what is “worthwhile knowledge” and to “clarify on whose behalf a study is conducted and, thus, for whom, in what ways, and why its contribution is important” (p. 16). Further, their recommendations for sport management researchers align with Sandberg and Alvesson (1) in that research should disrupt prevailing assumptions to build “new, significant, and meaningful knowledge that alters the way we understand and explain sport management practice” [(2), p. 16]. In the field of sport marketing specifically, Kim et al. [(3), p. 59] argue that sport marketing research has historically “been more normal research practice focusing on post-positivistic [consumer] behavior-based studies,” with the notion of “normal research” stemming from Kuhn's [(4), p. 163] reference to a “highly convergent activity based firmly upon a settled consensus acquired from scientific education and reinforced by subsequent life in the profession.” Kim et al. [(3), p. 59] highlight the need for “optimal balance between normal research with convergent thinking and innovative practices with divergent ideas” for scientific progress.

The purpose of this commentary is to serve as a point of departure for discussions on progressing the field of sport marketing in meaningful and impactful ways. We echo Stenling and Fahlén's (2) “call to arms” with an explicit focus on bridging sport marketing and critical social science. We agree with others who advocate for collapsing disciplinary silos [e.g., (5, 6)], and assert that sport marketing research has much to gain from engaging with critical social science assumptions, worldviews, and perspectives to examine complex issues in sport. We outline how sport marketing researchers may challenge the pre-existing assumptions in the field by encouraging others to “read ‘horizontally' to gain a ‘multi-silo' perspective of the phenomenon of interest, thereby facilitating the creation of knowledge that makes us think of phenomena in new ways” [(2), pp. 16–17]. In what follows, we discuss two research propositions, (1) consumer culture theory and (2) the circuit of culture, as two important frameworks that integrate sport marketing and critical social science. From the outset, we acknowledge that these are only two of many potential areas for synthesis, and even within these frameworks, there are numerous possibilities to explore. However, to help others envision our proposals, for each proposition we offer actionable recommendations as innovative research directions that promise new knowledge discovery.

Frisby [(7), p. 2] describes critical social science as “a way of empowering individuals by confronting injustices in order to promote social change.” Research that adopts critical approaches features prominently in sport sociology, with scholars suggesting that incorporating these perspectives into sport management research can advance the field (8). Researchers that utilize a critical view “are concerned about goals other than profit and with representing the interests of those affected by managerial actions, such as workers, athletes, volunteers, customers, marginalized populations, and the public at large” [(7), p. 6]. These approaches appear infrequently in journals within the domain of sport marketing, despite their importance in sport management research for “unpacking the less-desirable aspects of sport as a social system,” resulting in a stable foundation “upon which positive change in sport can be made” [(9), p. 9]. According to Sayer [(10), p. 768], the job of critical social science is “to ‘unsettle' existing academic ideas.” Therefore, incorporating critical social science approaches into sport marketing research may help to “expand our understanding of sport's role in society, how it may be an exclusionary space and to provide a strong theoretical basis for practical improvements by challenging power relations” [(9), p. 2].

Sport is a commodity and practice, and is a globally popular, highly visible, and influential part of society. Corporate brands use the appeal and excitement of sport to communicate their messages to consumers (11) and accomplish their business objectives (12). Currently there is momentum—and arguably a strong need—for professional sport (and related corporate partnerships) to have positive social impact, to help achieve social justice, and to minimize (the effects of) inequality. Arguably, sport marketing plays an equally important role in attracting large audiences to sport and associating corporate brands with the social and cultural values of sport. In this sense, marketing campaigns that activate and promote corporate partnerships with sport are “privileged form[s] of social communication” that can be utilized by marketers to influence culture, social interactions, and identities [(13), pp. 103–104]. Through marketing, brands create powerful stories and compelling narratives that consumers use to process their own tensions, desires, and anxieties that originate from broader societal problems (14). For example, Nike's “For Once Don't Do It” advertisement following the Black Lives Matter Movement, Amazon's Climate Pledge Arena in Seattle, and Scotiabank's “Hockey for All” campaign, to name a few. Yet, the “corporatization, privatization, and branding” of social justice issues by commercial organizations has become “increasingly complex, messy, and blurred” [(15), p. 523], with regards to corporate intentions and “rising consumer expectations of corporate social responsibility” [(16), p. 132]. As consumers become more discerning of corporate partnerships with sport, brands may be perceived as exploitative, disingenuous, and superfluous (17–20). Additionally, athletes and coaches, given their celebrity status and large followings, can “shape fans' attitudes, beliefs, and behaviors” [(21), p. 36], which can influence how sport marketers communicate with consumers. Crucially, framing sport marketing and consumer research from a position that upholds sport as a distinct social and cultural practice and queries *why* and *how* definitions and meanings of sport serve some interests over others [e.g., professional athlete, sport organization, corporate entity, fan/consumer; (22)], begins a long, convoluted journey to problematize and disrupt our assumptions about the association of corporate brands with sport.

## Consumer culture theory

Consumer Culture Theory (CCT) “refers to a family of theoretical perspectives that address the dynamic relationship between consumer actions, the culture marketplace, and cultural meanings” [(23), p. 868]. It consists of four salient, interconnected theoretical dimensions consisting of: “(1) consumer identity projects, (2) marketplace cultures, (3) the sociohistoric patterning of consumption, and (4) mass-mediated marketplace ideologies and consumers' interpretive strategies” [(23), p. 871]. According to CCT, supporters can create and attribute meaning to the images, texts, and objectives that are commercially produced and through which consumers make sense of their environment, which can lead to consumers redefining the meaning of a brand based on their collective interpretation thereof (14, 24). Thus, supporter groups may dictate the symbolism of brands associated with sport, with positive values (e.g., distinctiveness) benefitting the brand (25), and resistance occurring if they do not perceive that the team derives value from the connection (18). Rokka [(14), p. 114] recently commented that “CCT's future looks promising in its commitment and ability to foster critical, contextually sensitive, and reflexive cultural insights into marketing—an important foundation for marketing strategy and practices.” As such, CCT can play a role in exploring the contextual factors that influence how supporters and supporter groups interpret and attribute meaning to a brand.

At present, much sport marketing consumer research is largely informed by cognitive psychological (behavioral) or economic theories. While these studies are important for continued understanding of consumer attitudes toward products/services and purchasing behaviors, there is an opportunity to engage with CCT and social theories (e.g., social constructivism, feminism, critical race theory) to critique “the structural foundations and limitations of the consumers' experiential universe” [(26), p. 386]. CCT is often set within the context of social historical production as well as the prevailing socio-economic conditions, which contextualizes consumer-based practices and perspectives within the structures and systems that transcend lived experience. This allows “CCT researchers [to look] toward understanding market systems and dynamics and [approach] consumer culture not just as a matter of what consumers do but also how the world in which they do it is constituted” [(27), p. 135]. Ultimately, the goal is to give greater consideration to “the context of contexts” [(26), p. 396]. That is, to “pay increased attention to the contexts that condition consumption” [(26), p. 389]. Sport marketing research needs to, in the very least, consider how consumption experiences are embedded within broader social structures, cultural norms, and ideological injustices, including (among others): racism, gender relations, homophobia, and classism. These contexts cannot be ignored as consumers navigate, engage in, and challenge everyday consumer culture. “Looking at the ways that everyday consumption practices reproduce larger cultural and social frameworks is also a matter of asking not only how consumption is influenced by social forms and processes, but how it participates in the constitution of society” [(26), p. 396]. Given this discussion, we offer our first research proposition and four corresponding actionable recommendations.

*Proposition 1:* Critical engagement with CCT can provide a lens to examine realities beyond the individual sport consumer/fan.

*Actionable Recommendations:*

- Develop a better understanding of the meanings and values linked with signs, symbols, rituals, and traditions that shape brand community identity creation and development in sport.
- Generate a better understanding of the micro- and macro-level contextual influences (systemic and structural) of market and social systems that guide sport consumption experiences, identities, and communities.
- Seek a better understanding of the important actors (e.g., marketers, sport organization executives, brand executives) that participate in the contexts of sport consumption—actors that have their own social and cultural values.
- Establish a better understanding of ‘sport consumer-brand consumption' relationships as functions of sport, wherein both sport and consumption are recognized as social and cultural practices that can confer identity, values, and beliefs.

## The circuit of culture

Previous research on sport-related advertising and the communication of corporate brand partnerships in sport have adopted the circuit of culture as a framework to critically analyze sport-related promotion and advertising (28, 29). Accordingly, the circuit of culture, which traces the “lifecycle” of a commodity in contemporary society (30), has become an important component of research related to the growth of advertising, consumption, and commercialization within society (31). It consists of five interrelated cultural processes: production, representation, consumption, and identity, the components necessary to adequately examine commodities (30). As a circuit, the starting point is irrelevant, since the journey of explaining the meaning of an artifact involves analysis at each moment; cognizant that the processes are not distinct, rather each element converses with and blends into the next (30), overlapping one another and, hence, mutually defining and jointly dependent (32). It is the combined articulation or linkages of these processes that begin to explain the meaning cultural artifacts possess and the identities that they construct and/or embody (30). According to du Gay et al. [(30), p. 3], the five interlinked spheres facilitate the exploration of cultural artifacts in terms of “how it is represented, what social identities are associated with it, how it is produced and consumed, and what mechanisms regulate its distribution.”

In sport contexts, research often focuses more on the representation and identity components, and fails to examine consumption and regulation (31). Thus, this research captures more of the critical perspective that misappropriation and inaccurate representation can have on groups of people (28), while analyzing the individuals that are responsible for generating the advertisements (33). Conversely, sport marketing research often does not consider the content or sociocultural implications of advertisements (particularly on marginalized groups), nor that of the activations undertaken by brands involved with sport, instead focusing on the consumption of products (e.g., purchase intention), attitudinal outcomes (e.g., sponsor image), and other sponsorship constructs, including supporter identification, fit, and awareness (34, 35).

In critical social science research, sport-related advertisements are critically analyzed using the circuit of culture framework to examine the content, how the advertisement was produced, how the cultural intermediaries responsible for creating the advertisement chose to represent the sexuality, gender, and race of the people that appeared in the advertisement, and how the advertisement was consumed and interpreted by viewers (28). However, the focus of this research adopts the critical perspective without considering the marketing-related outcomes for the advertised brand (e.g., awareness, attitudes) after the advertisement has been processed and interpreted. Therefore, marketing research evaluating corporate brand partnerships in sport should incorporate critical analysis of the production, identity, and consumption contained within the circuit of culture to move beyond the individual consumer into the broader societal and cultural context surrounding the positioning of the advertised brand. Given this discussion, we offer our second research proposition and four corresponding actionable recommendations.

*Proposition 2:* The circuit of culture offers a framework to explore how consumers react to and interpret the content of marketing campaigns that activate and promote corporate partnerships in sport.

*Actionable Recommendations:*

- Examine how advertising content and messaging influences consumers' subsequent attitudinal and behavioral responses to the advertisements.
- Explore the power relations between cultural intermediaries and marginalized populations and any resulting social injustices.
- Investigate how marketers' idealized and/or stereotypical representations of particular groups (e.g., men/women, racial, Indigenous, LGBTIQ2S+, etc.) can impact a group's identity and consumption behaviors.
- Analyze the manner in which a product's intended meaning is altered through consumption experiences and identity regulation.

## Conclusion

In this commentary, we propose two research propositions that connect sport marketing and critical social science research. These are unorthodox but imperative proposals that require radical reconsideration of two research fundamentals. First, these propositions challenge traditional ontological marketing worldviews that embrace a (post)positivist paradigm (3), and instead advocate for ontological claims that “reality is created through [macro and] microsocial interactions” (interpretivist paradigm) and/or “reality is rooted in the tensions surrounding historically entrenched power relations” (critical realist paradigm; [(7), pp. 2–3]. Second, these propositions prompt the need for new research designs (e.g., ethnography) that necessitate the integration of qualitative or multi/mixed methods, which diverts from the “normal research” [(4), p. 163] that is generally conducted in sport marketing. Taken together, we uphold that these propositions open up possibilities for sport marketing research to be innovative and impactful, to disrupt repeated and institutionalized lines of reasoning/inquiry, and to create new expectations for what is worthwhile knowledge in the field.

## Data availability statement

The original contributions presented in the study are included in the article/supplementary material, further inquiries can be directed to the corresponding author/s.

## Author contributions

ZE was responsible for conceptualizing and writing the manuscript. SG and TE offered guidance on the conceptualization and writing of the manuscript, in addition to edits, revisions, and helping to re-write some parts of the final manuscript. All authors have made a substantial, direct, and intellectual contribution to this manuscript and approved the manuscript in the form in which it was submitted.

## Conflict of interest

The authors declare that the research was conducted in the absence of any commercial or financial relationships that could be construed as a potential conflict of interest.

## Publisher's note

All claims expressed in this article are solely those of the authors and do not necessarily represent those of their affiliated organizations, or those of the publisher, the editors and the reviewers. Any product that may be evaluated in this article, or claim that may be made by its manufacturer, is not guaranteed or endorsed by the publisher.
